# Expediting clinician assessment in the diagnosis of autism spectrum disorder

**DOI:** 10.1111/dmcn.14530

**Published:** 2020-04-02

**Authors:** Matthew J Sanchez, John N Constantino

**Affiliations:** ^1^ Department of Psychiatry School of Medicine Case Western Reserve University Cleveland OH USA; ^2^ University Hospitals Cleveland Medical Center Cleveland OH USA; ^3^ Departments of Psychiatry and Pediatrics Washington University in St Louis St Louis MO USA; ^4^ St Louis Children’s Hospital St Louis MO USA

## Abstract

**Aim:**

To investigate a novel observational rating protocol designed to expedite clinical diagnosis of autism spectrum disorder (ASD).

**Method:**

Two hundred and forty patients referred to a tertiary autism center (median age 8y 9mo, range 2y 6mo–34y 8mo; 188 males, 52 females) were rated using an adaptation of the Childhood Autism Rating Scale, Second Edition (CARS‐2) based exclusively on patient observation (CARS‐2^obs^). Scores were compared to expert diagnosis of ASD, parent‐reported Social Responsiveness Scale, Second Edition (SRS‐2) and, in a selected subset of patients, the Autism Diagnostic Observation Schedule, Second Edition (ADOS‐2).

**Results:**

CARS‐2^obs^ distinguished patients with a clinical diagnosis of ASD from those with non‐ASD neuropsychiatric disorders (mean score=18 vs 11.7, *p*<0.001). Severity ratings on the CARS‐2^obs^ correlated with the ADOS‐2 (*r*=0.68, *ρ*=0.64) and SRS‐2 (*r*=0.31, *ρ*=0.32). A CARS‐2^obs^ cutoff point equal to or greater than 16 demonstrated 95.8% specificity and 62.3% sensitivity in discriminating individuals with ASD from individuals without ASD in a specialty referral setting.

**Interpretation:**

The CARS‐2^obs^ allows the rapid acquisition of quantitative ratings of autistic severity by direct observation. Coupled with parent/teacher‐reported symptoms and developmental history, the measure may contribute to a low‐cost diagnostic paradigm in clinical and public health settings, where positive results might help reduce delays in diagnosis, and negative results could prompt further specialty assessment.

**What this paper adds:**

- The Childhood Autism Rating Scale, Second Edition based on patient observation distinguished individuals with versus without autism spectrum disorder (ASD).
- A score equal to or greater than 16 on this assessment showed high specificity for a diagnosis of ASD.

## What this paper adds

- The Childhood Autism Rating Scale, Second Edition based on patient observation distinguished individuals with versus without autism spectrum disorder (ASD).
- A score equal to or greater than 16 on this assessment showed high specificity for a diagnosis of ASD.

This article is commented on by Fein on page 772 of this issue.

**Editor's Choice**

Late detection of autism spectrum disorder (ASD) delays access to appropriate management. My Editor's choice for the July issue describes a new tool for trained clinicians to observe and rate children's behaviours in order to rapidly discriminate between those with and without ASD. In conjunction with increased awareness and service optimization, such approaches based on brief observation of relevant features by professionals may meet currently unmet needs.

Video Podcast: https://youtu.be/lUChbsEWhps

**Abbreviations**
ADOS‐2: Autism Diagnostic Observation Schedule, Second Edition
ASD: Autism spectrum disorder
CARS‐2: Childhood Autism Rating Scale, Second Edition
RRB: Restricted, repetitive behavior
SCI: Social Communication and Interaction
SRS‐2: Social Responsiveness Scale, Second Edition

Autism spectrum disorder (ASD) is estimated to affect 1 in 59 children in the general population.^1^ ASD diagnosis is exclusively clinical, although frequent discoveries are being made in elucidating its complex genetic and neurological basis.^2^ Key components of diagnosing ASD include a positive developmental history, current symptoms, symptoms that cannot be better explained using an alternative diagnosis, and observational confirmation using DSM‐5 criteria.^3^ Patients are typically referred to specialized providers who use standard clinical measures to diagnose and differentiate ASD from other disorders. Traditional measures, such as the Autism Diagnostic Observation Schedule, Second Edition (ADOS‐2) or Autism Diagnostic Interview Revised, employed by most autism specialists are time‐consuming, require extensive training, and are performed by specialized providers.^4^, ^5^ Additionally, some studies have suggested that both the ADOS‐2 and Autism Diagnostic Interview Revised should be performed together because they better reflect a best estimate diagnosis of ASD.^6^ This can result in substantial delay between the time when a child is first suspected of having ASD, is referred to a particular provider, completes the diagnostic process, and finally receives management/therapy services. Parents have reported visiting, on average, four to five clinicians en route to an ASD diagnosis and a significant number have reported dissatisfaction and distress with the process.^7^, ^8^, ^9^ Such delays contribute to the fact that, across the USA, the average diagnosis is in the fourth year of life, even though symptoms are present in most affected children before the age of 2 years. Minimizing the time between initial suspicion of ASD and treatment is critical to maximizing the impact of interventions for this disorder.^10^

Although traditional ‘expert‐dependent’ rating systems represent critical tools for the evaluation of some patients with ASD, they themselves have been validated against clinician diagnosis. For many patients with ASD, diagnosis is well within the scope of practice and experience for doctoral‐level clinicians without specialized training in the use of autism diagnostic measures. A more direct approach to diagnostic confirmation could potentially reduce costs and time to intervention.^11^ A clinician‐implemented rating scale that assists in both reliable diagnostic confirmation and characterization of severity^12^ could serve as a necessary complement to parent‐ and teacher‐reported rating scales that together represent a comprehensive appraisal of the ASD symptom burden, with the added benefit of tracking change over time within clinical settings.^13^, ^14^, ^15^, ^16^

The Childhood Autism Rating Scale, Second Edition (CARS‐2) is a previously validated and extensively implemented tool for assessing the ASD symptom burden.^17^, ^18^, ^19^ It has the benefit of relatively minor requirements for training and implementation, which is made possible by narrative scoring anchors for quantifying every assessed dimension of symptomatology. The CARS‐2 is traditionally scored using multiple inputs including parent and teacher reporting, records, and clinical observation. A previous study described a modified implementation of the CARS‐2 using a time‐limited direct observation (CARS‐2^obs^), which standardized the scoring inputs. In a pilot study, implementation revealed a strong correlation between untrained clinician ratings and Autism Diagnostic Interview Revised scores with high interrater reliability.^14^ In this study, we extend these initial findings in a large clinical population of children referred for ASD diagnosis and treatment. The aim of the study was to validate the ability of the CARS‐2^obs^ to rapidly discriminate between patients with ASD and patients without such a diagnosis. Given the number of children with ASD, improvements in diagnostic efficiency could result in large overall healthcare system benefits, particularly in public and telehealth settings.

## Method

### Sample

The sample included a consecutive series of patients referred to the Washington University Autism Clinical Center for whom a standardized protocol scored using the CARS‐2 was implemented as a clinical standard in the initial patient encounter (see the Measures section of the article). Two hundred and forty‐one patients completed the CARS‐2 assessment (Fig. S1, online supporting information). One patient was excluded due to unconfirmed scoring. Ages ranged from 2 years 6 months to 34 years 8 months (median 8y 9mo, 11 patients ≥18y). After the CARS‐2, each patient had two 90‐minute clinical evaluations that incorporated standardized ratings and the ADOS‐2 to aid in the diagnosis if, at the end of the evaluation, the clinician was not yet confident in their diagnosis. Two hundred and twenty‐three patients completed the Social Responsiveness Scale, Second Edition (SRS‐2) and 19 patients completed the ADOS‐2. Notably, the clinician performing the final diagnostic evaluation was not the same clinician who administered the CARS‐2^obs^ scoring; they were blinded from the CARS‐2^obs^ results when making their final diagnosis. Physicians who were experts in the diagnosis of autism gave final diagnoses in all cases. This protocol was reviewed by the Washington University Human Research Protection Office Institutional Review Board and qualified for waiver of individual informed consent based on being conducted primarily as part of a clinical program evaluation to determine the validity of measurement methods in a clinical context.

A total of 167 patients were diagnosed with ASD; 102 were scored using the standard CARS‐2, 64 were scored with the high‐functioning type of the CARS‐2, and one was scored with an unknown measure. Of the 73 patients without ASD, attention‐deficit/hyperactivity disorder (ADHD) was the primary diagnosis for 35 (48%). Other common diagnoses were anxiety disorders, unspecified mood disorder, and expressive/mixed language disorder. Patient characteristics, including relevant behavioral rating scales, are described in Table 1. There were no differences in sex, age, and ethnicity between groups. Three patients did not have ethnicity recorded and one patient did not have cultural lineage recorded. There was no between‐group difference regarding mean Child Behavior Checklist internalizing and externalizing scores. Mean Adaptive Behavior Assessment System General Adaptive Composite, Adaptive Behavior Assessment System Social Affect scores, and SRS‐2 total scaled scores were significantly different between groups (Table 1).

**Table 1:** Characteristics of study sample population (*n*=240)

	ASD (*n*=167)^a^	Non‐ASD (*n*=73)^b^	*p*
Males (%)	135 (81)	53 (72)	*p*=0.1542
Median age (IQR), y:mo	9:3 (5:2–14:1)	8:3 (5:8–12:8)	T *p*=0.298, KW *p*=0.50
2–4y	22	6	–
4–6y	26	13	–
6–10y	40	25	–
10–15y	46	22	–
15–18y	25	4	–
>18y	8	3	–
White (%)	136 (81)	54 (75)	*p*=0.222
Non‐Hispanic ethnicity (%)	163 (98)	71 (100)	*p*=0.254
Mean CBCL externalizing T score (SD)	63.1 (11.6)	65.0 (13.6)	T *p*=0.361
Mean CBCL internalizing T score (SD)	64.5 (9.6)	65.2 (13.7)	T *p*=0.683, KW *p*=0.201
Mean ABAS GAC (SD)	69.0 (13.8)	77.1 (12.5)	T *p*<0.001, KW *p*<0.001
Mean ABAS Social Affect (SD)	69.5 (13.1)	78.0 (11.5)	T *p*<0.001, KW *p*<0.001
Mean CARS‐2^obs^ (SD)	18.0 (5.2)	11.7 (2.8)	T *p*<0.001, KW *p*<0.001
Mean SRS‐2 total T score (SD)	77.4 (9.8)	70.2 (12.7)	T *p*<0.001, KW *p*<0.001

^a^ Standard, *n*=102; high‐functioning, *n*=64; unknown measure, *n*=1. ^b^Attention‐deficit/hyperactivity disorder, *n*=35; other, *n*=38. IQR, interquartile range; ASD, autism spectrum disorder; T, *t*‐test; KW, Kruskal–Wallis test; CBCL, Child Behavior Checklist; ABAS, Adaptive Behavior Assessment System; GAC, General Adaptive Composite; CARS‐2^obs^, Childhood Autism Rating Scale, Second Edition based on patient observation; SRS‐2, Social Responsiveness Scale, Second Edition.

### Measures

We implemented an adaptation of the CARS‐2 based exclusively on a brief protocol‐based observation during the first patient encounter (CARS‐2^obs^). Nurse practitioners and early career physicians conducted the observations for the CARS‐2^obs^ scoring. The scale has two versions: standard and high‐functioning for patients who are verbally fluent with an estimated IQ greater than 80 or developmental age equal to or greater than 6 years.^20^ The CARS‐2 traditionally consists of 14 behavior domains with a 15th domain assessing general impressions. Each domain is scored on a 7‐point scale from 1 to 4 (with mid points), with a total score of 15 to 60. The CARS‐2 is traditionally scored using information from multiple sources; however, for our cohort, the CARS‐2 was scored exclusively based on a 15‐minute in‐person observation protocol as the first element of clinical assessment in each case (Appendix S1, online supporting information). The standard or high‐functioning CARS‐2 scoring sheet was completed immediately. All evaluators previously completed training on the standard CARS‐2, which included training manual review and completion of a practice assessment. Evaluators also completed two trial ratings based on viewing a sample of 15‐minute patient videos that demonstrated interrater mean score reliabilities of 0.76 (mild cases) and 0.93 (severe cases). Otherwise, evaluators had no previous formal training on this protocol. The first eight items of the CARS‐2 quantify behaviors that define autistic syndromes and have been supported as differentiating individuals with autism from individuals without such a diagnosis. We combined these first eight items into a single composite score, referred to as CARS‐2^obs^.

The SRS‐2 is a validated 65‐item scale for use by caregivers and/or teachers that quantifies observed autistic traits and symptoms over the past 6 months.^21^, ^22^ These were completed by either parent, usually the mother. T scores were used in all analyses except for correlation analysis, where raw scores were used since they provided a broader range of scoring and were more suitable for comparison given that many patients with elevated CARS‐2^obs^ scores had a scaled SRS‐2 T score of 90 (maximum allowable).

The ADOS‐2 is a semi‐structured standardized assessment of communication, social interaction, and play.^5^ All ADOS‐2 assessments were completed by examiners with research‐level certification in the measure. ADOS‐2 Social Affect, restricted, repetitive behaviors (RRBs), and total raw scores were used in the analyses.

### Data analysis

SAS v4 (SAS Institute, Cary, NC, USA) or SPSS v25 (IBM Corp., Armonk, NY, USA) were used to perform all statistical analyses. Two tailed Student’s *t*‐tests compared the total CARS‐2^obs^ and SRS‐2 scores. Internal consistency testing was performed. Analysis of variance with Tukey–Kramer adjustment compared CARS‐2^obs^ and total SRS‐2 between patients with ASD scored with the standard CARS‐2, patients with ASD scored with the high‐functioning CARS‐2, patients with ADHD, and other patients with other diagnoses. Pearson correlation coefficients compared CARS‐2^obs^ with raw SRS‐2 RRBs, Social Communication and Interaction (SCI) and total scores; raw ADOS‐2 Social Affect and RRB and total scores; and raw SRS‐2 SCI and RRB scores, which correlated highly in past studies. *p‐*values are reported throughout. For distributions that violated assumption of normality testing using the Shapiro–Wilk statistic, we included non‐parametric testing (Kruskal–Wallis test for comparison and Spearman’s rho [*ρ*] for correlation) whenever appropriate, which produced similar results. Distributions that deviated from assumptions of normality may have done so because the sample was clinically ascertained; therefore, in cases where non‐parametric tests were implemented, we also reported the results from parametric testing. Linear regression analysis was performed for CARS‐2^obs^ with raw SRS‐2 and ADOS‐2 total scores with *R* ^2^ values reported, along with lines of best fit, 95% confidence intervals and 95% prediction limits. Regression analyses were verified for validity, including homoscedasticity and normal distribution of residuals. We examined the receiver operating characteristic curve of the CARS‐2^obs^ against clinician diagnosis of ASD along with the corresponding Youden cutoff point. Total SRS‐2 and CARS‐2^obs^ scores were compared to clinician diagnoses of ASD for sensitivity and specificity. Sensitivity, specificity, as well as positive and negative likelihood values were generated manually using Microsoft Excel.

## Results

### CARS‐2^obs^ distribution and comparison

**Figure 1:**
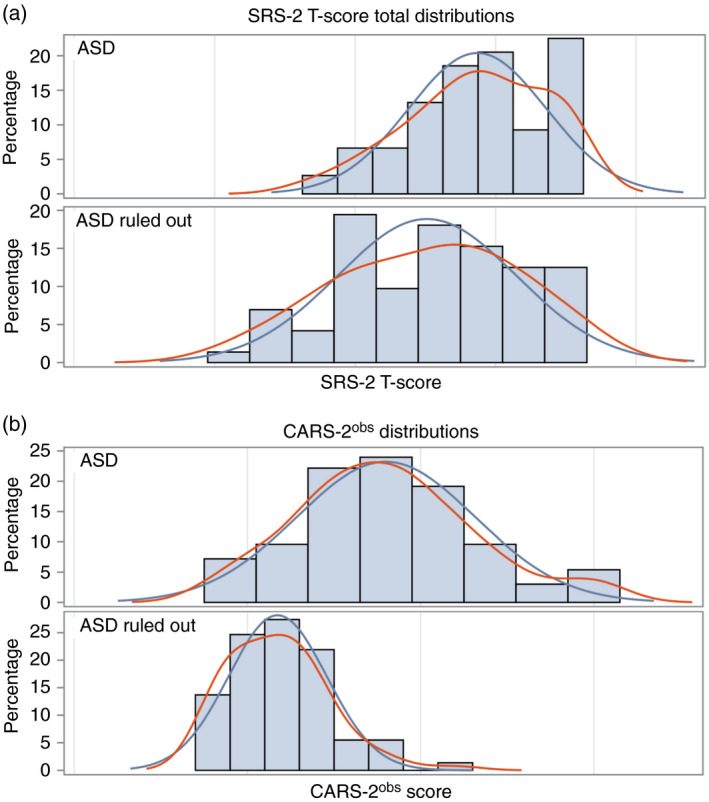
(a) Scaled Social Responsiveness Scale, Second Edition (SRS‐2) total score distribution comparison between patients diagnosed with autism spectrum disorder (ASD) and patients without such a diagnosis (ASD ruled out). (b) Childhood Autism Rating Scale, Second Edition based on patient observation (CARS‐2^obs^) score distribution comparison between patients with ASD and patients without (ASD ruled out). All patients had been referred to the Autism Clinical Center for suspicion of ASD and were ultimately diagnosed with ASD or another primary diagnosis.

Figure 1 shows the scaled SRS‐2 and CARS‐2^obs^ distributions respectively. The ASD and non‐ASD groups exhibited unimodal, continuous distributions. CARS‐2^obs^ mean scores were significantly different between patients with ASD and patients without this diagnosis (18 vs 11.7, all testing *p*<0.001; Table 1). Notably, there was a 2 SD difference between the ASD and non‐ASD groups on the CARS‐2^obs^, whereas the SRS‐2 scores differed by only 0.5 SD. Reliability testing showed that individual items on the CARS‐2^obs^ were highly internally consistent (*α*=0.913). No significant difference was found in CARS‐2^obs^ scores between standard and high‐functioning ASD CARS‐2 or ADHD and all other diagnoses. There were significant differences between any ASD group and any non‐ASD group, supporting the notion that the CARS‐2^obs^ distinguishes between patients with ASD and patients without such a diagnosis and does not differentiate between subgroups (Table S1, online supporting information). Since there were no significant differences in CARS‐2^obs^ test forms, we did not distinguish between forms going forward. Comparison of items 10 to 14 on the original CARS‐2 forms between groups showed that, overall, patients with ASD had higher scores (Fig. S2, online supporting information).

### Correlation of SRS‐2 and ADOS‐2 with CARS‐2^obs^

**Figure 2:**
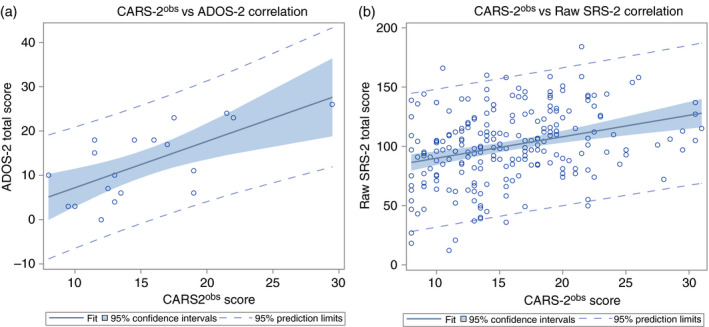
(a) Regression plot of the Childhood Autism Rating Scale, Second Edition based on patient observation (CARS‐2^obs^) and Autism Diagnostic Observation Schedule, Second Edition (ADOS‐2) for all patients. (b) Regression plot of the CARS‐2^obs^ and raw Social Responsiveness Scale, Second Edition (SRS‐2) total scores for all patients. The line of best fit is noted along with the 95% confidence intervals and 95% prediction limits. Although there is evidence of overestimation for a CARS‐2^obs^ greater than 25, suggesting that the relationship may not be uniformly linear, removal of this group of individuals did not significantly affect the model.

Comparison of CARS‐2^obs^ with the ADOS‐2 Social Affect, ADOS‐2 RRB, and ADOS‐2 total scores showed strong positive correlations throughout (Pearson Social Affect *r*=0.64, *p*=0.032; RRB *r*=0.69, *p*=0.011; total *r*=0.68, *p*=0.015; Spearman Social Affect *ρ*=0.61, *p*=0.006; RRB *ρ*=0.62, *p*=0.004; total *ρ*=0.64, *p*=0.003). Linear regression showed a linear correlation with an *R* ^2^ of 0.458. Figure 2 is a scatterplot of these results, showing a line of best fit and 95% confidence intervals/prediction limits.

Comparison of CARS‐2^obs^ with raw SRS‐2 SCI, raw SRS‐2 RRB, and raw SRS‐2 total scores showed moderate positive correlations throughout (Pearson SCI *r*=0.32, RRB *r*=0.28, total *r*=0.31, all *p*<0.001; Spearman SCI *ρ*=0.32, RRB *ρ*=0.32, total *ρ*=0.32, all *p*<0.001). Linear regression showed a linear correlation with an *R* ^2^ of 0.095 (Fig. 2b). A group of patients fell below the line of best fit at CARS‐2^obs^ values greater than 25. The analysis indicated that this group did not significantly impact the overall model. SRS‐2 SCI and SRS‐2 RRB subscale scores correlated highly in this sample (*r*=0.75, *p*<0.001), as observed previously.

### **CARS‐2 and SRS‐2 scoring for ASD diagnosis**

**Figure 3:**
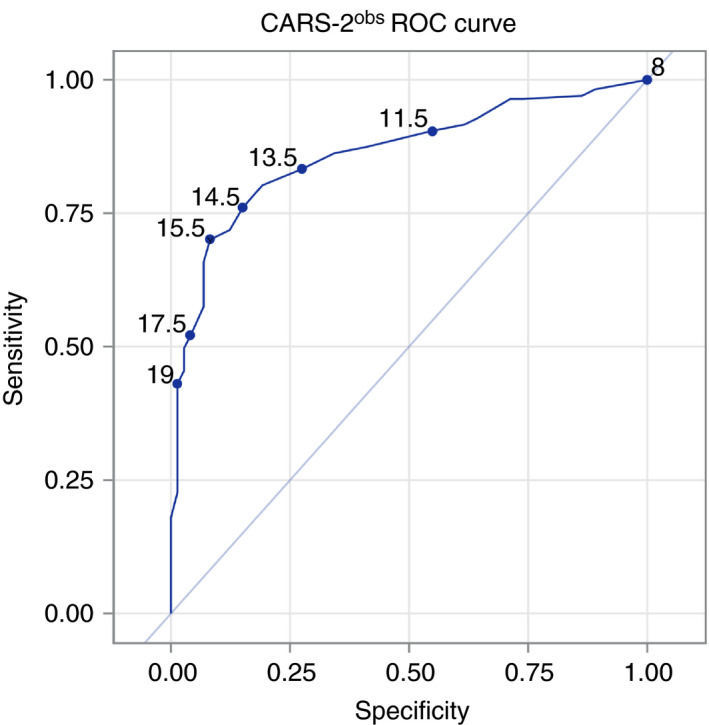
Receiver operating characteristic curve calculated for the total Childhood Autism Rating Scale, Second Edition based on patient observation (CARS‐2^obs^) score. Individual tested cutoff points with corresponding scores are noted on the graph.

Partial correlations controlling for other variables when comparing CARS‐2^obs^ versus ASD diagnosis showed minimal impact of these variables on the correlation (Table S2, online supporting information). Scores were compared to clinician diagnosis to determine sensitivity, specificity, as well as positive and negative likelihood ratios. In this sample, the receiver operating characteristic curve for CARS‐2^obs^ showed a remarkable level of convergence with expert diagnosis, with an area under the curve of 0.86 (Fig. 3). The Youden cutoff point for a CARS‐2^obs^ of 16 (rounded up from 15.5) gave a specificity of 93.2% and sensitivity of 65.9%. The maximally (100%) sensitive cutoff point was 8 and the maximally specific cutoff point was 23. Multilevel likelihood ratios showed a range of positive likelihood ratio from 1.12 to 30.79 and negative likelihood ratios from 0.13 to 0.69 depending on the cutoff (Table S3, online supporting information).

When the total SRS‐2 T scores were included, an SRS‐2 equal to or greater than 61 and CARS‐2^obs^ equal to or greater than 16 produced a specificity of 95.8% and sensitivity of 62.3%, with a positive likelihood ratio of 14.83 and negative likelihood ratio of 0.39. Higher cutoffs resulted in greater decreases in sensitivity than increases in specificity. Incremental validity testing showed SRS‐2 T scores adding 0.016 to the *R* ^2^ for CARS‐2^obs^ versus ASD diagnosis (*R* ^2^=0.453 vs 437, *p*=0.054).

## Discussion

This study demonstrates the utility of a rapid, standardized implementation of the CARS‐2 in the evaluation of children suspected of having ASD. CARS‐2^obs^ scores exhibited a 2 SD difference between patients with ASD and patients without such a diagnosis in a tertiary referral center. CARS‐2^obs^ scores were highly correlated with ADOS‐2 scores and were moderately correlated with SRS‐2 scores. The stronger correlation with ADOS‐2 versus SRS‐2 scores is consistent with previous findings, which showed that instruments using the same measure (e.g. observation vs parental report) provide similar information about symptom burden.^13^ These data strongly support the notion that standardized clinician ratings based on brief observations can be used to differentiate between patients with ASD and patients without.

Previous studies attempted to define cutoffs for the traditional CARS‐2 form, but they are not currently incorporated into the official CARS‐2 scoring system.^17^ A CARS‐2^obs^ score equal to or greater than 16 rendered high specificity with acceptable sensitivity for ASD in this cohort. Although sensitivity is not as high as specificity at this cutoff point the selection of a cut point may vary to optimize either purpose. All patients in this cohort had high suspicion for ASD or positive screening testing. An elevated score would aid a clinician in making a diagnosis and shorten lag time between suspicion, diagnosis, and access to services. Children for whom clinicians cannot confidently confirm an ASD diagnosis using the CARS‐2^obs^ should be referred to a specialist for further evaluation. Currently, many children, even those strongly suspected of having ASD, are referred to specialists. Clinicians may also want to trade off sensitivity versus specificity in varying situations. To aid with this, multilevel likelihood ratios are provided in Table S3 for varying cutoff scores. These results support the utility of the CARS‐2^obs^ in assisting clinicians with the rapid clinical confirmation of a diagnosis of ASD.

When combined with an SRS‐2 T score cutoff equal to or greater than 61, specificity increased. This is consistent with a previous study, which found that an SRS‐2 T score of 60 (raw score 58–60) on teacher‐ and parent‐reported SRS‐2 resulted in a specificity of 96% for ASD.^13^ We observed a positive predictive value of 97% using a CARS‐2^obs^ equal to or greater than 16 and an SRS‐2 equal to or greater than 61. SRS‐2 scores explained only a small amount of additional variance and added 1.6% to *R* ^2^ when combined with CARS‐2^obs^; this is consistent with the data showing that adding SRS‐2 increases specificity by a relatively small amount of 2.5%.

Obtaining a positive developmental history, determining the presence of current symptoms consistent with ASD (most reliable when endorsed by multiple informants), and clinician confirmation, including ruling out an alternative developmental/psychiatric diagnosis that could better explain the symptoms, are three key pillars for establishing a diagnosis of ASD.^23^ The CARS‐2 includes ratings of disorders that most commonly mimic the presentation of autism: 10, anxiety; 11 and 12, specific language impairment; 13, ADHD; and 14, intellectual disability. When markedly elevated, these items can alert clinicians to possible competing or comorbid diagnoses. In our study, the group clinically diagnosed with ASD on average had higher scores in these domains than the group not diagnosed with ASD, suggesting comorbidity with elevated scores rather than a primary diagnosis of another disorder. Individually high scores on these non‐core ASD symptom items should prompt consideration of a comorbid versus alternative diagnosis and performance of further assessment or psychometric testing.

One limitation of this study is that the cutoffs and likelihood ratios cited earlier were derived from our sample, a referral population to an autism center. This resulted in a rigorous test of the measure’s ability to discriminate cases from non‐cases; however, the results regarding sensitivity cannot be directly extrapolated to the general population. Nevertheless, the emphasis of utilizing this instrument for the purpose of clinician confirmation provides a method that relates to the only true standard existing for diagnostic assignment, that is, confirmation by a clinician. Further investigation is needed to examine the performance of CARS‐2^obs^ in cross‐validation samples and varying clinical settings. Another limitation was the relatively modest number of children who had ADOS‐2 scores. However, validation of all autism diagnostic measures rests on expert clinician diagnosis, and in this study psychiatrists and pediatricians who were experts in the diagnosis of autism provided all final diagnoses. Nonetheless, the fact that a minority of patients had ADOS‐2 performed may mean that these patients were not fully representative of the sample; additional studies exploring this correlation could investigate if this holds true with larger sample sizes. This study also had relatively low numbers of females and children with intellectual disability.

These findings support the CARS‐2^obs^ as a promising tool for one of the three pillars of the diagnostic process for ASD diagnosis, especially in settings where referral to autism specialists may be unfeasible or cause significant delays. CARS‐2^obs^ scores differentiated between individuals with and without ASD and exhibited strong correlations with scores on the ADOS‐2. Further studies should be performed to validate these findings in other populations and determine if implementation of this methodology in primary care, telehealth, and public health settings can complement the rapid acquisition of data on developmental history and symptoms from parent and teacher informants as part of a feasible and reliable approach to diagnosing ASD, especially for children who have clear symptoms of the disorder. These data reinforce the conclusion of a previously published report that standardized clinician ratings based on brief observations, without needing extensive rater training, show tremendous promise as a rapid and cost‐effective approach that could empower clinicians to assess patients for ASD.

## Supporting information


**Figure S1:** Flow of participants through study.

**Figure S2:** CARS‐2 item 10 to 14 score distributions between patients with and without ASD.

Table S1: Significance of CARS‐2obs scoring difference between ASD groups and other diagnoses.

Table S2: Partial correlation between CARS‐2obs scores and ASD diagnoses controlling for variables.

Table S3: Multi‐level positive and negative likelihood ratios for respective CARS‐2obs score cutoffs.

Appendix S1: Written protocol for standardized CARS2obs assessment.
