# Supplementary figures and images for: Expediting clinician assessment in the diagnosis of autism spectrum disorder
Source: Dev Med Child Neurol. 2020 Apr 2;62(7):806–12. doi: 10.1111/dmcn.14530 (PMC7540056; doi:10.1111/dmcn.14530)

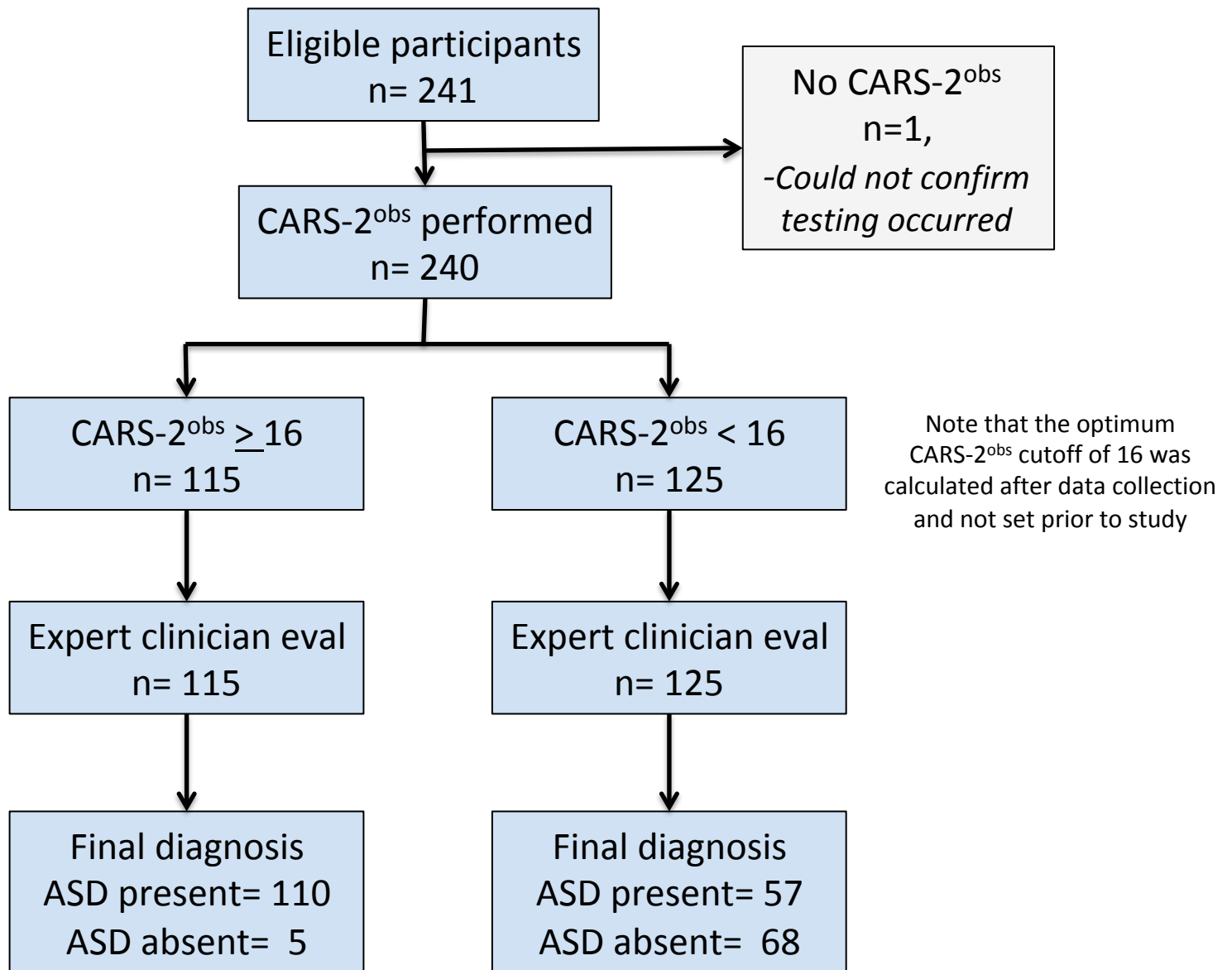

Figure S1. Diagram illustrating flow of participants through study.

Supplement: Supplementary file 1 — Figure S1: Flow of participants through study. [file DMCN-62-806-s001.pdf]
