# Supplementary material for: Expediting clinician assessment in the diagnosis of autism spectrum disorder
Source: Dev Med Child Neurol. 2020 Apr 2;62(7):806–12. doi: 10.1111/dmcn.14530 (PMC7540056; doi:10.1111/dmcn.14530)
Supplement: Supplementary file 2 — Figure S2: CARS‐2 item 10 to 14 score distributions between patients with and without ASD. [file DMCN-62-806-s002.pdf]

Figure S2. CARS-2 item 10-14 score distributions between ASD patients and non-ASD patients (ASD ruled out). The competing diagnoses that each item helps to assess for are listed below. Item 10: Anxiety, Items 11 and 12: Language delay, Item 13: ADHD, Item 14: Intellectual disability.

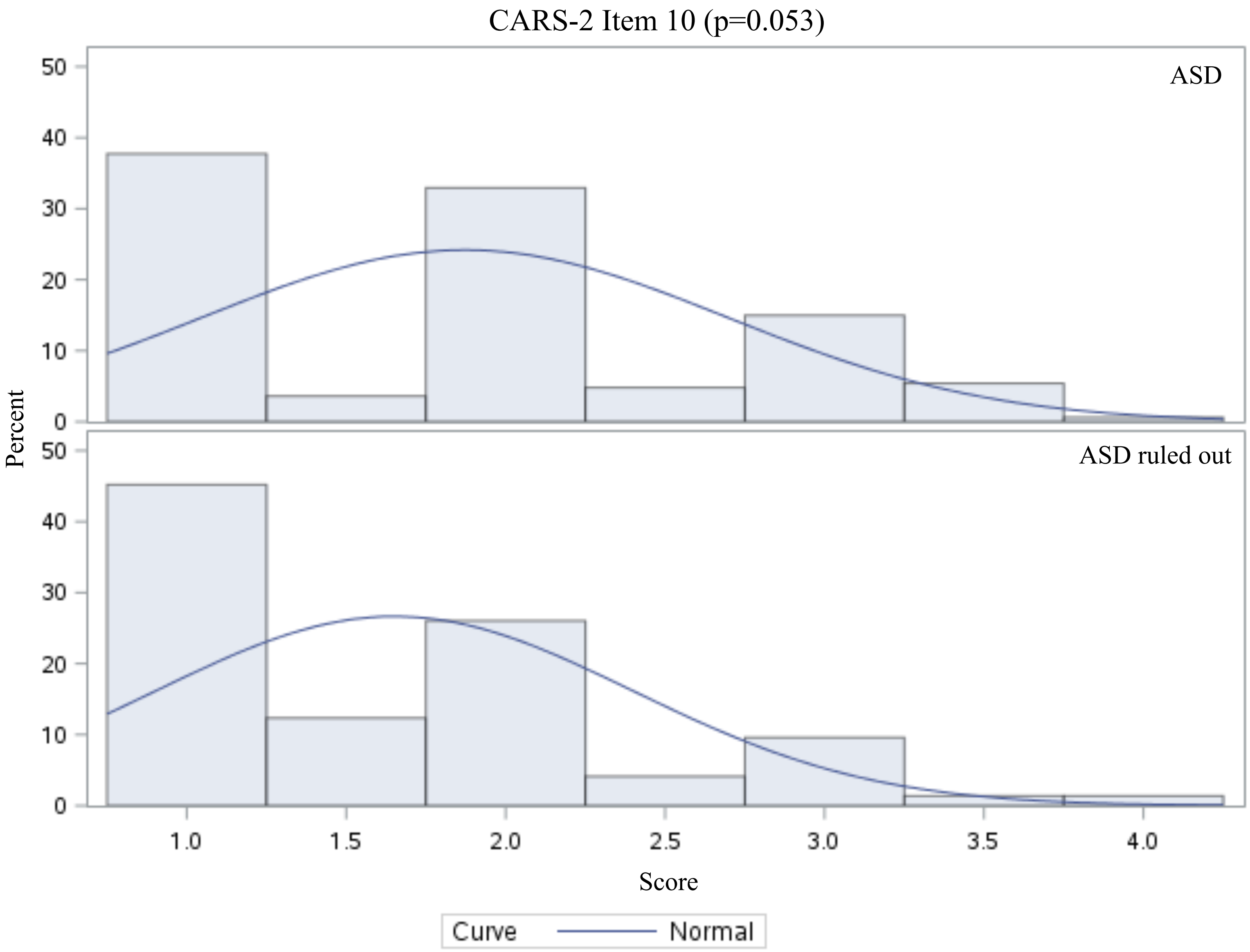
CARS-2 Item 10 (p=0.053)
ASD
ASD ruled out
Percent
50
40
30
20
10
0
1.0
1.5
2.0
2.5
3.0
3.5
4.0
Score
Curve
Normal

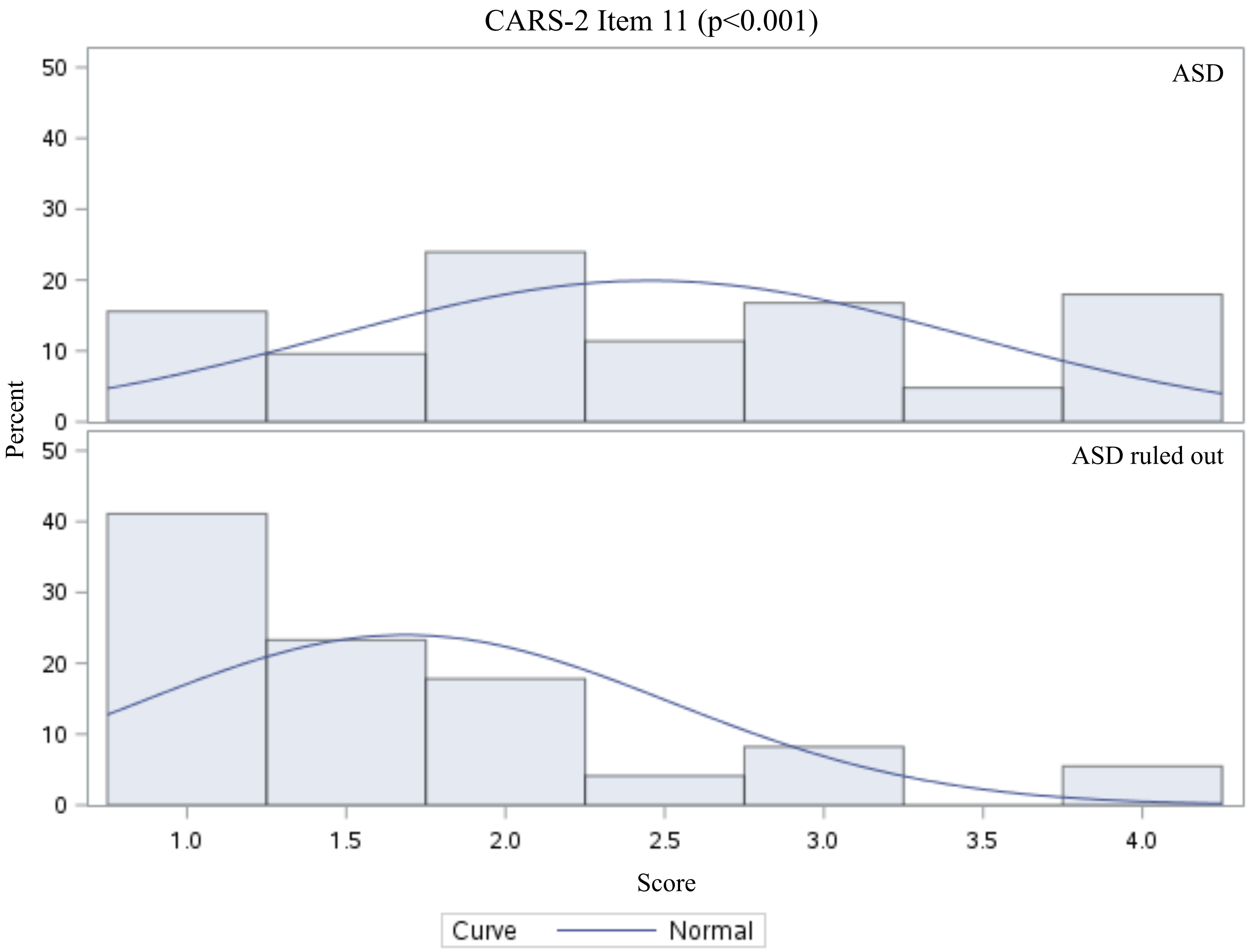

CARS-2 Item 11 (p<0.001)
ASD
ASD ruled out
Percent
0
10
20
30
40
50
1.0
1.5
2.0
2.5
3.0
3.5
4.0
Score
Curve
Normal

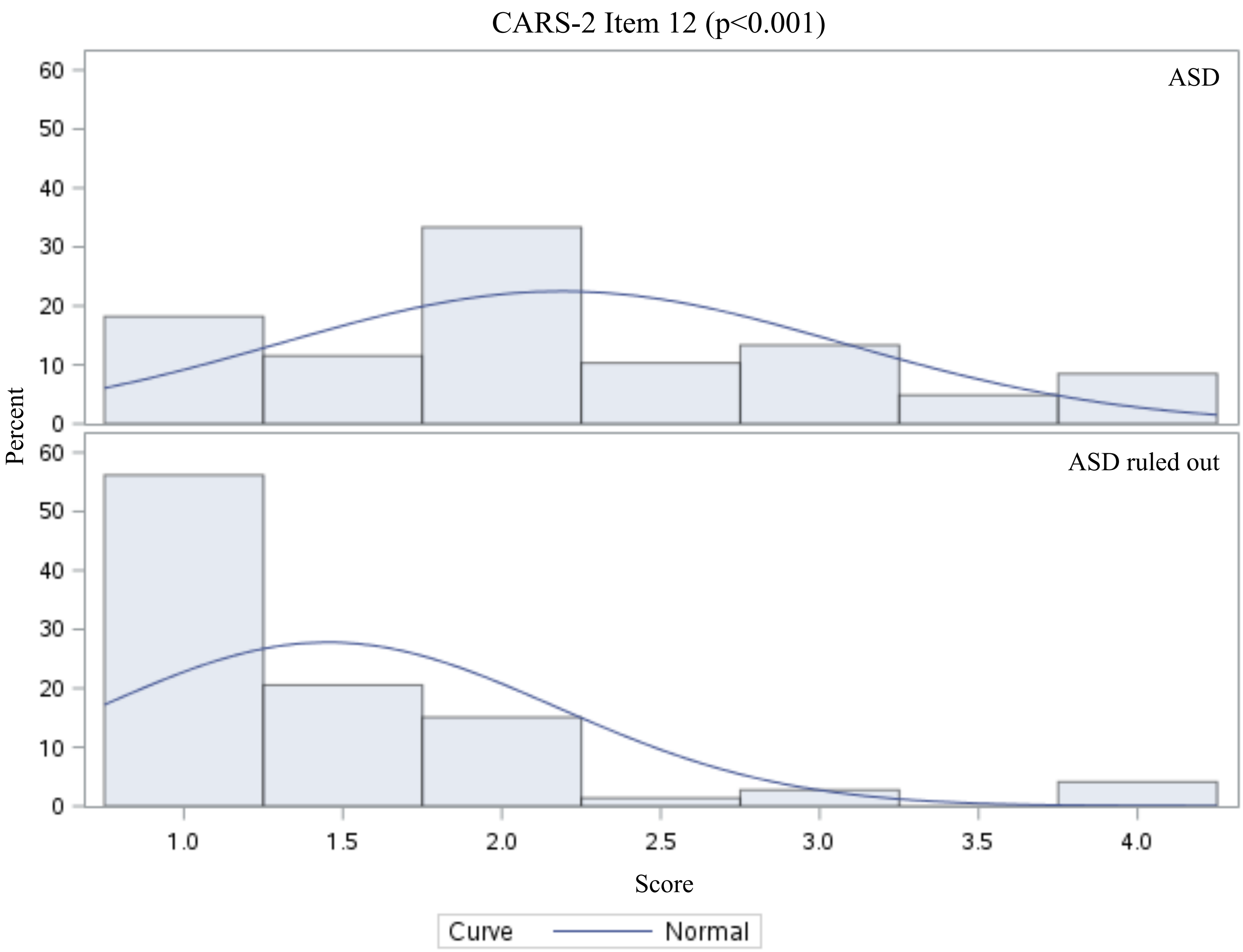
CARS-2 Item 12 (p<0.001)
ASD
ASD ruled out
Percent
0
10
20
30
40
50
60
1.0
1.5
2.0
2.5
3.0
3.5
4.0
Score
Curve
Normal

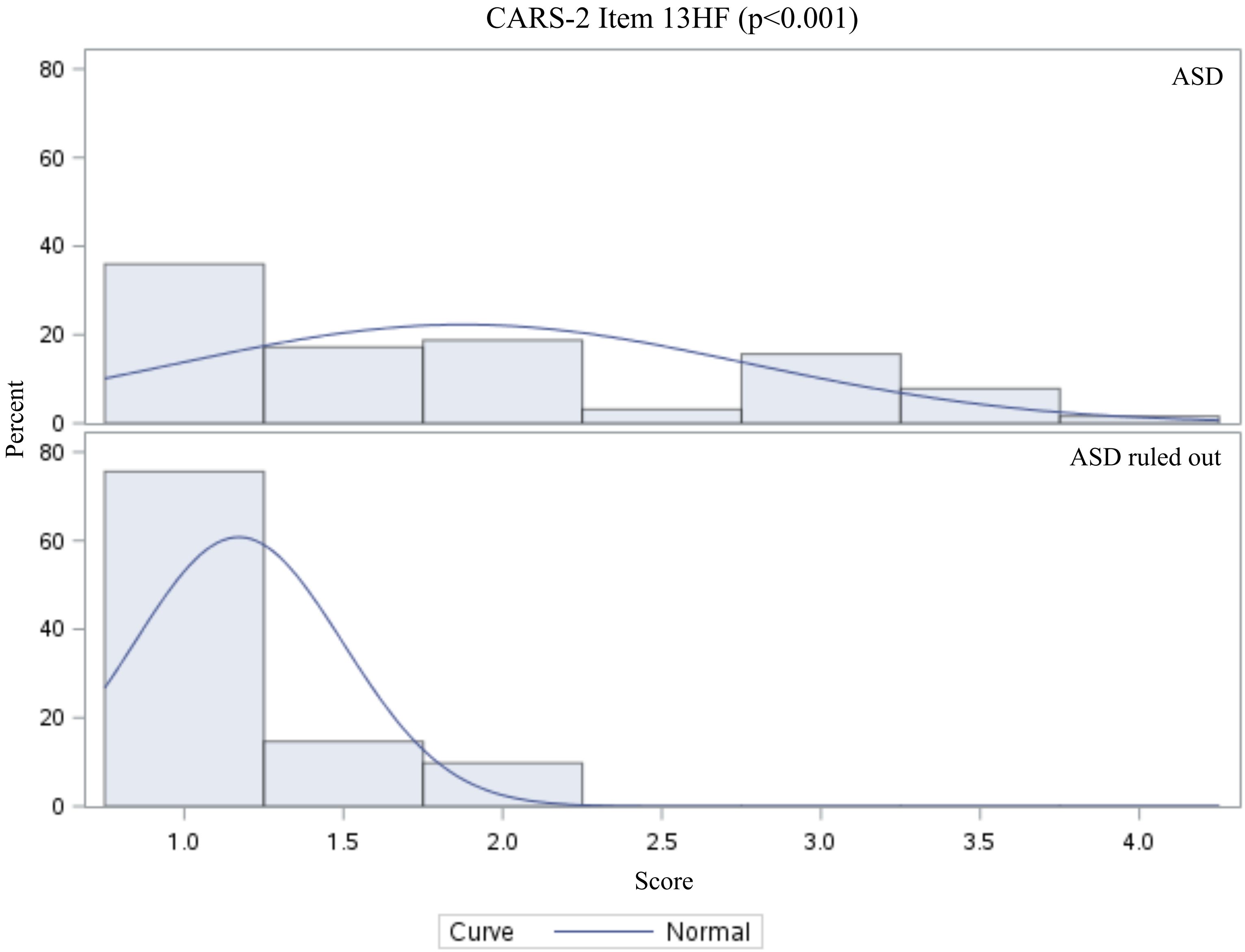
CARS-2 Item 13HF (p<0.001)
ASD
ASD ruled out
Percent
0
20
40
60
80
Score
1.0
1.5
2.0
2.5
3.0
3.5
4.0
Curve
Normal

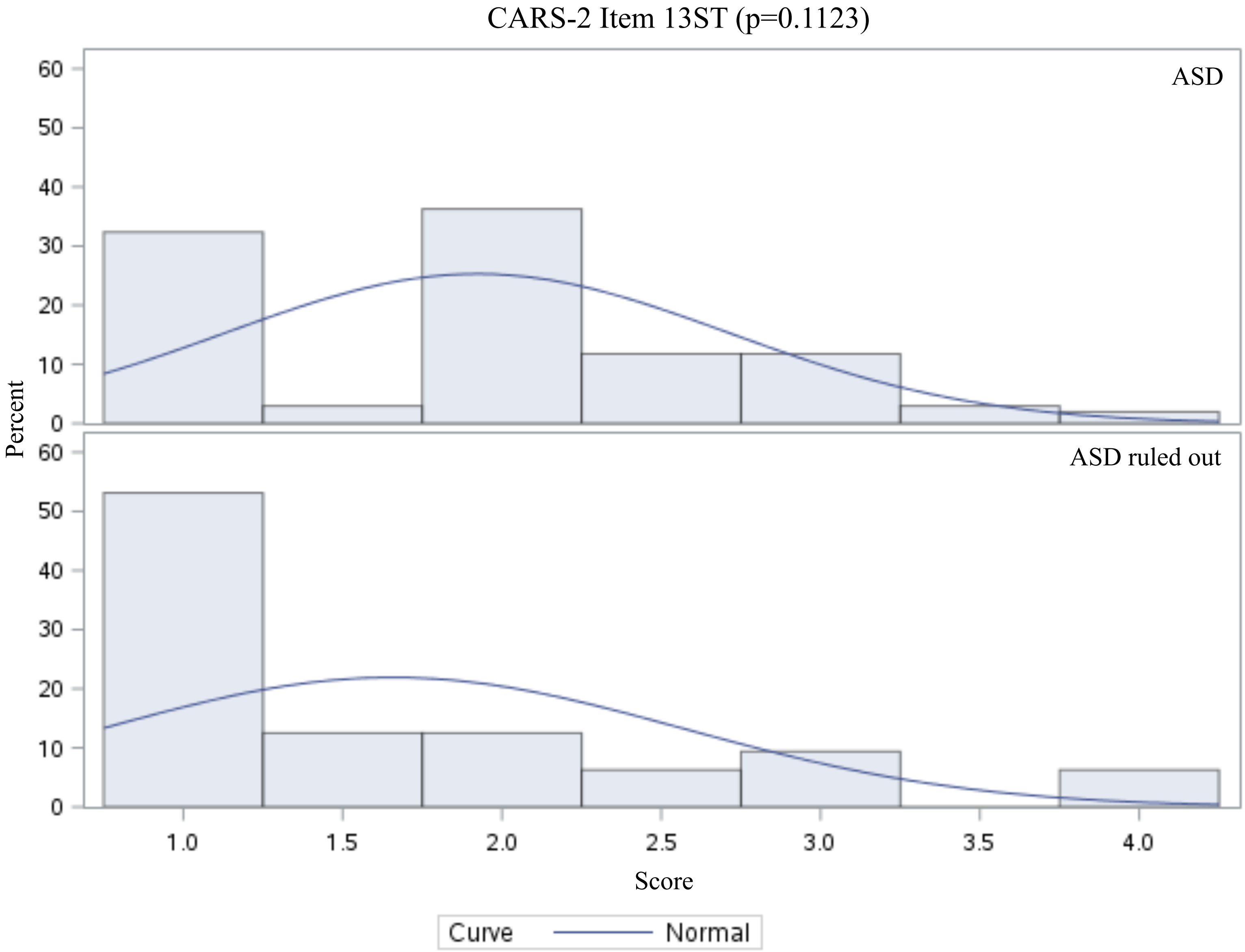
CARS-2 Item 13ST (p=0.1123)
ASD
ASD ruled out
Percent
60
50
40
30
20
10
0
1.0
1.5
2.0
2.5
3.0
3.5
4.0
Score
Curve
Normal

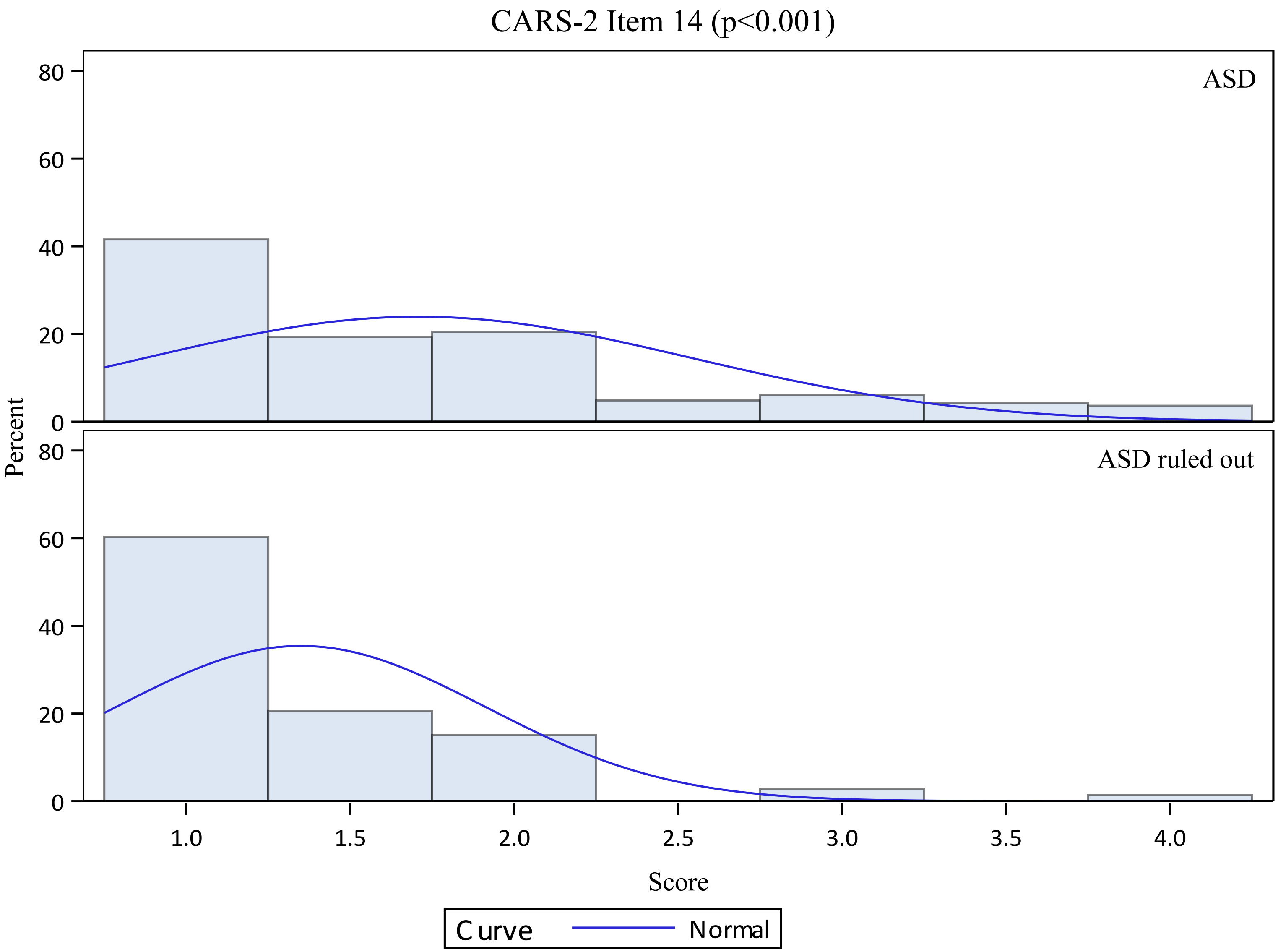
CARS-2 Item 14 (p<0.001)
ASD
ASD ruled out
Percent
0
20
40
60
80
1.0
1.5
2.0
2.5
3.0
3.5
4.0
Score
Curve
Normal
