# Supplementary material for: Expediting clinician assessment in the diagnosis of autism spectrum disorder
Source: Dev Med Child Neurol. 2020 Apr 2;62(7):806–12. doi: 10.1111/dmcn.14530 (PMC7540056; doi:10.1111/dmcn.14530)
Supplement: Supplementary file 3 — Table S1: Significance of CARS‐2obs scoring difference between ASD groups and other diagnoses. [file DMCN-62-806-s003.docx]

Table S1. Significance of CARS-2obs scoring difference between ASD groups and other diagnoses. Individual boxes represent p values when comparing the CARS-2obs score of the two patient categories.

|  | CARS-2^obs^ score |  |  |  |
| --- | --- | --- | --- | --- |
|  | ASD-CARS2-ST | ASD-CARS2-HF | ADHD | Other |
| ASD-CARS2-ST |  | 0.9801 | <0.0001 | <0.0001 |
| ASD-CARS2-HF | 0.9801 |  | <0.0001 | <0.0001 |
| ADHD | <0.0001 | <0.0001 |  | 0.8253 |
| Other | <0.0001 | <0.0001 | 0.8253 |  |
