# Supplementary material for: Expediting clinician assessment in the diagnosis of autism spectrum disorder
Source: Dev Med Child Neurol. 2020 Apr 2;62(7):806–12. doi: 10.1111/dmcn.14530 (PMC7540056; doi:10.1111/dmcn.14530)
Supplement: Supplementary file 4 — Table S2: Partial correlation between CARS‐2obs scores and ASD diagnoses controlling for variables. [file DMCN-62-806-s004.docx]

Table S2. Partial correlation between CARS-2obs scores and ASD diagnoses controlling for variables noted below.

|  | n | Correlation (r) | Correlation (ρ) | p-value |
| --- | --- | --- | --- | --- |
| CARS-2^obs^ and ASD | 240 | 0.533 | 0.577 | <0.0001 |
| **Control variable** |  |  |  |  |
| with Age | 240 | 0.531 | 0.576 | <0.0001 |
| with Sex | 240 | 0.530 | 0.574 | <0.0001 |
| with Race | 239 | 0.537 | 0.580 | <0.0001 |
| with Ethnicity | 237 | 0.532 | 0.574 | <0.0001 |
| with ABAS GAC | 199 | 0.498 | 0.537 | <0.0001 |
| with ABAS Social | 204 | 0.478 | 0.517 | <0.0001 |
| with CBCL INT T-score | 163 | 0.586 | 0.624 | <0.0001 |
| with CBCL EXT T-score | 163 | 0.584 | 0.625 | <0.0001 |
| with SRS total T-score | 223 | 0.483 | 0.531 | <0.0001 |
| with all variables above | 135 | 0.506 | 0.537 | <0.0001 |
