# Supplementary material for: Expediting clinician assessment in the diagnosis of autism spectrum disorder
Source: Dev Med Child Neurol. 2020 Apr 2;62(7):806–12. doi: 10.1111/dmcn.14530 (PMC7540056; doi:10.1111/dmcn.14530)
Supplement: Supplementary file 5 — Table S3: Multi‐level positive and negative likelihood ratios for respective CARS‐2obs score cutoffs. [file DMCN-62-806-s005.docx]

Table S3. Multi-level positive and negative likelihood ratios for respective CARS-2obs score cutoffs.

| CARS-2^obs^ score | Positive Likelihood Ratio | Negative Likelihood Ratio |
| --- | --- | --- |
| > 9 | 1.12 | 0.22 |
| > 10 | 1.35 | 0.13 |
| > 11 | 1.49 | 0.22 |
| > 12 | 1.81 | 0.21 |
| > 13 | 2.52 | 0.21 |
| > 14 | 4.18 | 0.25 |
| > 15 | 5.85 | 0.32 |
| > 16 | 9.69 | 0.37 |
| > 17 | 8.46 | 0.46 |
| > 18 | 18.4 | 0.52 |
| > 19 | 30.8 | 0.58 |
| > 20 | 23.1 | 0.69 |
