# Supplementary material for: Expediting clinician assessment in the diagnosis of autism spectrum disorder
Source: Dev Med Child Neurol. 2020 Apr 2;62(7):806–12. doi: 10.1111/dmcn.14530 (PMC7540056; doi:10.1111/dmcn.14530)
Supplement: Supplementary file 6 — Appendix S1: Written protocol for standardized CARS2obs assessment. [file DMCN-62-806-s006.docx]

**Appendix S1. Written protocol for standardized CARS2obs assessment.**

Observation

The procedure outlined below should be utilized to administer the CARS-2 assessment in an abbreviated, 15 min observation session. Using only this 15 min session (and basic demographic information), the clinician will be able to score all items outlined in the CARS-2 assessment. This will provide a strong diagnostic evaluation related to the presence of traits characteristic of Autism Spectrum Disorder in patients.

Clinicians should complete either the Standard CARS-2 (CARS-2-ST) or a modified version for higher functioning individuals (CARS-2-HF). Criteria for which assessment to administer are included below:

- CARS-2 ST should be used for any patients who are not verbally fluent, patients 6 years or younger, or those with below average intelligence.
- CARS-HF should be used for any verbally fluent patients who are 6 years or older and average to above average intelligence.

The 15-m session should be broken down into three, 5 min subsections. This guide is meant to promote efficient and semi-standardized assessment administration. It is *not* meant as an exact protocol, and there is no script that you must follow. The clinician should have some freedom to provide the assessment as they see fit. Please score the assessment immediately following the 15 min observation session. See “Scoring” section for further instructions on scoring the CARS-2.

Conversation or Free Play

Assessment of this subsection consists of verbal or nonverbal interaction with the patient to the greatest extent possible. For children who are verbal, engage the child in basic conversation (i.e. how old they are, questions about school, etc.). This time should also be used to observe if/how the child initiates conversation with you and how they communicate with their caregiver.

For children who are non-verbal, observe how child communicates (i.e. gestures, signs, augmentative communication, etc.) as well as how the child explores materials in the room. Ascertain whether or not the child is capable of turn taking (Can they toss the ball back and forth with you? Can they take turns shaking the maraca?)

Symbolic Interactive Play

Use materials from doll house and CARS-2 kit (if age appropriate) to observe spontaneous and imitative/prompted play. If materials are not appropriate due to age or interest level, have the patient recount a personal event from the past.

- Symbolic Play
  - Talking on toy phone
  - Playing with doll house figures, baby doll, or toy animals (not just self- stimulation)
- Symbolic Interactive Play
  - Child talks on toy phone with examiner
  - Playing with doll house figures, baby doll, or toy animals with the examiner
  - Using action figures to interact with the examiner
- Pretend and Imaginative Play
  - Child uses play materials to conduct a novel story
  - Acting out a scene on a toy phone or doll house

Sensory Stimulation

For the remaining 5 minutes, transition to a sensory activity such as:

- Blowing up a balloon and then releasing it
- Blowing bubbles

Observe patient’s affect, initiation of joint attention, shared enjoyment, requesting, and any unusual sensory behaviors or repetitive mannerisms. If bubbles or balloons do not produce a sensory reaction, and you believe one may be elicited, the following materials may also be used:

- Loud noise on phone or computer
- Music
- Turning lights on and off
- Having patient talk about their favorite things (interests, topics, etc.)

Scoring

Complete the CARS-2 scoring. Ignore the CARS-2 scoring page. Enter specific item tallies into medical record as specified on ACC evaluation template.
